# Privacy Policy Compliance of Mobile Sports and Health Apps in China: Scale Development, Data Analysis, and Prospects for Regulatory Reform

**DOI:** 10.2196/73651

**Published:** 2026-02-11

**Authors:** Rengui Guo, Fanhong Chen

**Affiliations:** 1 School of Law, Central South University, No.932, Lushan South Road, Yuelu District, Changsha, 410012, China, 86 18810373347

**Keywords:** sports and health apps, personal data, sensitive information, privacy policy compliance, personal information protection, regulatory reform

## Abstract

**Background:**

Driven by technological advancements, the proliferation of mobile sports and health apps has revolutionized health management by improving efficiency, cost-effectiveness, and accessibility. While the widespread adoption of these platforms has transformed public health practices and social well-being in China, emerging evidence suggests that inadequacies in their privacy policies may compromise personal information (PI) protection.

**Objective:**

This study aimed to conduct a systematic evaluation of privacy policy compliance among 286 mobile sports and health apps in the Chinese Mainland, benchmarking them against the Personal Information Protection Law and associated PI regulatory guidelines.

**Methods:**

This study develops a privacy policy compliance indicator scale based on the information life cycle and the legal framework for PI protection in the Chinese Mainland. This scale consists of 5 level 1 indicators and 37 level 2 indicators that assess the privacy policy compliance.

**Results:**

The privacy policy compliance of 286 sports and health apps generally performed worse, with only a minimal number (n=11, 3.8%) of apps scoring above 90 points (rated as excellent), nearly half (n=121, 42.3%) of apps scored below 60 points (rated as unqualified). Among the 5 level 1 evaluation indicators for privacy compliance in sports and health apps, the compliance rate for PI collection (mean 74%, SD 25.8%) is the highest, while the compliance rate for PI storage (mean 53.5%, SD 28.4%) is the lowest. The compliance rates for privacy policies across the remaining 3 level 1 evaluation indicators, such as PI usage (mean 54.2%, SD 24.4%), PI entrusted processing, sharing, transferring, and disclosing (mean 62.2%, SD 19.8%), and PI security and feedback (mean 61.7%, SD 21.3%), fall around 60%. Out of 37, 17 level 2 evaluation indicators show a compliance rate below 60%. The compliance rate with privacy policies for 5 level 2 evaluation indicators is exceptionally high, including collection subject (mean 97.2%, SD 16.5%), collection type (mean 99%, SD 10.2%), collection purpose (mean 96.2%, SD 19.3%), reasons for sharing, transferring, and disclosing PI (mean 91.6%, SD 27.8%), and feedback channel (mean 93.4%, SD 24.9%). Notably, 3 indicators exhibit compliance rates below 20%, including sensitive information storage (mean 14%, SD 34.7%), constraints of automatic decision-making (mean 9.4%, SD 29.3%), and deceased user rule (mean 5.2%, SD 22.3%). Authorization for sensitive information (mean 29.4%, SD 45.6%) lagged behind general information (mean 83.6%, SD 37.1%).

**Conclusions:**

Although some apps have established commendable policies, there are gaps that compromise the efficacy of PI protection. Considering this, this paper proposes targeted actions for 3 stakeholders: users, regulators, and legislators. Only through coordinated action can the app ecosystem close the compliance gaps, reduce PI protection risks, and restore user trust in digital services.

## Introduction

With the popularity of mobile phones and people’s increasing interests in sports and health management [1-3], mobile sports and health apps, which are closely related to physical exercise and provide exercise knowledge, assist with physical activities, track workout data, and support health management, have emerged and are constantly growing [3-7]. Currently, the app stores on the 2 major mobile operating systems, Android (Google) and Apple iOS, offer a vast array of apps related to health monitoring [8,9], calorie management [10-12], fitness guidance [13,14], health consultation [15,16], and other sports and health services [17]. These apps have enhanced the efficiency, cost-effectiveness, and accessibility of sports and health management, enabling people who are concerned about health through sports to learn exercise programs in real-time, track their diet, and improve their overall health condition using the apps [18-21]. It is reported that the extensive usage of these apps has significantly enhanced the social and public health landscape in China [22].

However, the rapid growth of mobile sports and health apps poses a threat to users’ personal privacy and data security [23-26]. Streaking privacy, which occurs when sports and health apps collect, process, and use personal exercise and physical health information without authorization, has caused serious legal issues and attracted widespread social attention around the world [27-29]. For instance, the US Federal Trade Commission ordered the Easy Healthcare Corporation to pay a $100,000 civil penalty for its Premom app collecting and sharing a significant amount of users’ personal information (PI) with third-party advertisers in violation of privacy protection, eavesdropping, and other applicable laws and regulations [30]. A Norwegian nonprofit organization has discovered that 10 of the most popular apps on Google Play Store, including sports and health apps, transmit users’ sensitive PI to third parties without their permission [31]. In China, these challenges are more acute. In December 2018, China’s Cyberspace Administration carried out a special regulatory action targeting numerous apps, including those related to sports and health, and discovered that 3496 apps were illegally collecting and using PI [32].

As a response, many countries, including the United States [33,34] and the European Union [35-37], have enacted bills to strengthen PI protection [38]. The United States has enacted laws such as the Health Insurance Portability and Accountability Act [39], the Health Breach Notification Rule [40], and the Children’s Online Privacy Protection Rule [41], which stipulate the obligation of health care organizations to safeguard health data, require health care organizations to establish a consent mechanism for sharing patients’ health data with third parties, outline the data breach notification mechanism for health care organizations and the legal responsibilities for disclosing patients’ sensitive data [42]. The European Union protects PI and privacy in a unified manner through the General Data Protection Regulation, grants 8 rights to users, and requires data processors to process personal data under the individual consent mechanism [43].

In response to the risks of PI leakage and privacy abuse posed by sports and health apps, China’s PI protection legal system adopts an approach [44], aiming to protect users’ privacy data and promote market development [27]. In 2019, the Cyberspace Administration of China made policies, such as methods for determining illegal collection and use of personal information by apps and provisions on the scope of necessary personal information for common types of mobile internet applications, to regulate the collection and use of PI by apps [45]. In light of the noncoercive nature of these policies and in view of the urgent need for PI protection [46], China enacted the Personal Information Protection Law (PIPL) in 2021 [47]. While public reports held that the PIPL has established a basic legal framework for PI collection, processing, and usage [48-50], academic scholars still argue that the PIPL should be significantly improved [51-54]. Meanwhile, since December 2021, the National Technical Committee 260 on Cybersecurity of Standardization Administration of China has successively made voluntary national standards, such as the Practical Guide to Cybersecurity Standards—Guidelines for the Classification and Grading of Network Data [55], the Information Security Technology - Personal Information Security Engineering Guidelines (PI Guidelines) [56], Information Security Technology—Implementation Guidelines for Notices and Consent in Personal Information Processing [57], and Information Security Technology—Guide for De-identifying Personal Information [58] to classify and grade PI for protection. Additionally, the Ministry of Industry and Information Technology of China regularly reports and requires rectification of apps that violate users’ rights and interests [59]. According to public reports, a total of 297 apps were required to rectify their illegal collection of PI in 2024 [60].

Under the above legal requirements, mobile sports and health apps, which collect and use users’ PI as the premise of their normal operation, need to establish an internal compliance management system for personal privacy and data security [44]. In this system, developing privacy policies stands in the foreground, because the formulation and implementation of privacy policies ensures that the apps’ market operation behavior meets the requirements of laws and regulations, thereby achieving a balance between internal self-discipline and external supervision. As required by PIPL and PI Guidelines, a legitimate and valid privacy policy is comprised of the collection, storage, use, entrusted processing, sharing, transfer, disclosure, consultation, and feedback of PI [61,62]. Accordingly, this paper attempts to measure these aspects of mobile sports and health apps’ privacy policy because the degree of PI protection is correlated with how well the app’s privacy policy conforms with PIPL and PI Guidelines.

There is a well-established body of work on privacy policies and PI protection in existing studies. For example, Tangari et al [4] explored free fitness apps on the Google Play Store in Australia and found that 88% of the apps will potentially collect user data. Parker et al [63] investigated and rated medicine-related apps for the Android mobile platform available in the medical store category of Google Play in the United Kingdom, United States, Canada, and Australia. Grundy et al [64] explored the risks posed by rated medicines-related apps when sharing user data. Alfawzan et al [65] emphasized the privacy policies of 23 most popular apps on women’s health in the Apple operating system and the Android system. Shipp and Blasco [66] assessed the privacy practices of a set of 30 Android menstruation-related apps that track users’ reproductive cycle, sex life, and health. Although research on the privacy policy compliance of mobile health (mHealth) apps is expanding, studies specifically examining the privacy policy compliance of mHealth apps closely related to sports still require further in-depth investigation. Scholars have conducted comparative in-depth research on the privacy policy compliance of China’s mHealth apps [67-69], focusing primarily on mobile mental health [29,70,71], mobile hospitals [28,72,73], contact tracing [74,75], traditional Chinese medicine treatments [76], and chronic disease management [22,77]. Research on the privacy policy compliance of Chinese mobile sports and health apps remains limited. Current research on privacy policy compliance for mobile sports and health apps is limited to a few jurisdictions, with a significant lack of attention still directed toward the privacy policy compliance issues of Chinese mobile sports and health apps. Currently, China boasts an exceptionally large user base for mobile sports and health apps. These apps collect users’ PI, particularly sensitive data, such as gender, location, exercise routes, heart rate, workout duration, weight, and exercise frequency. If the PI collected by these mobile sports and health apps is not gathered, used, and shared in a lawful, transparent, and trustworthy manner, it not only jeopardizes users’ personal privacy and safety but also poses a threat to national data security. Therefore, privacy policy compliance for mobile sports and health apps requires thorough analysis in the Chinese Mainland.

In order to comprehensively evaluate the compliance rate of privacy policies of sports and health apps in the Chinese Mainland, this paper deploys scenario-based contextual analysis [78] and information life cycle theory [79]. Nissenbaum [78] argued that the contextual integrity theory should serve as the cornerstone of privacy protection, because different contextual frameworks shape the norms of information flow across various scenarios, and these norms determine the legitimacy of information processing. There are typically 2 types of PI protection scenarios. The first type vertically categorizes different types of information based on the varying levels of processing risks, and the other type horizontally focuses on the different stages of information processing. Regarding the former, both domestic and international legislation generally adopt a dichotomy to differentiate between sensitive PI and general PI. This paper also follows this classification to evaluate their respective levels of protection compliance. For the latter, combining with the information life cycle stipulated in the PIPL, this study proposes 5 stages of PI collection, PI storage, PI usage, PI entrusted processing, sharing, transferring, and disclosing, and PI security and feedback, and further uses them as 5 primary indicators in the scale.

This study uses the legal framework for PI protection to develop a privacy policy compliance indicator scale and evaluate the compliance degree of privacy policies of 286 sports and health apps. In the Methods section, we focus on selecting, collecting, and analyzing sample apps, developing a compliance indicator scale, and designing the scoring process. In the Results section, we examine the level of PI protection of each privacy policy and report the results of the compliance assessment. In the Discussion section, we summarize the overall landscape and existing shortcomings in privacy policy compliance among sports and health apps and propose regulatory reforms to strengthen PI protection.

This study aims to (1) select, collect, and analyze privacy policies of sports and health apps developed for users in the Chinese Mainland, (2) establish a privacy policy compliance indicator scale based on the PIPL and PI Guidelines [22,27] (3) evaluate the compliance level of privacy policies of sports and health apps by scoring each indicator, and (4) provide suggestions on how to improve PI protection in the Chinese Mainland. This study enhances the discourse on balancing personal information protection with sustainable innovation in sports and health apps. By underscoring the necessity of enhancing privacy policy compliance and offering improvement suggestions, it offers insights for policymakers, developers, operators, and users across various nations regarding PI protection.

## Methods

### Study Design

We first comprehensively collected 714 apps from the app stores of Google Android and Apple iOS, the 2 mobile phone operating systems in the Chinese Mainland, between August 2 and August 6, 2025. Then we proceeded to examine the program description of apps to review their features and locate their privacy policies on August 6, 2025. Subsequently, we downloaded the privacy policies of the remaining apps, eliminating those that either did not allow a privacy policy download or lacked a privacy policy altogether between August 7 and August 8, 2025. Finally, we analyzed the compliance level of privacy policies of 286 valid sports and health apps with the PIPL and PI Guidelines and further proposed improvement suggestions regarding the gaps identified in the process of compliance evaluation from August 10 to August 20, 2025.

### Sample Selection and Inclusion Criteria

This study focused on the privacy policy compliance of sports and health apps. We selected the apps from the app stores of Android and Apple, the 2 popular mobile operating systems in the Chinese mainland. We opened the app stores (Yingyongshangdian in Chinese) section, selected the function of app category (Fenlei in Chinese), and put in keywords “sports and health” (Yundong Jiankang in Chinese) to filter apps. Through the comprehensive collection and search methods described above, we have gathered the initial sports and health sample apps. Then, we carefully reviewed the feature descriptions of each of the initial sports and health sample apps we collected, while simultaneously downloading their privacy policy documents. The apps included in the sample must meet the following 2 criteria. First, they must be explicitly designed for a diverse user base to offer sport guidance, real-time exercise data tracking, fitness education, and other sports and health services, and second, they must be intended for ordinary individuals rather than for sports and health management organizations or governments. The apps with the following criteria were excluded from the sample. First, the app provides only low-fat food options, recipes, and dietary control recommendations. Second, the app provides only psychological issue diagnosis, stress relief, and emotional support functions. Third, the app provides only sleep aid and sleep quality monitoring functions. Fourth, the app solely collects physical data, analyzes health issues, and provides fitness recommendations. Fifth, the app exclusively offers exercise instruction content and videos, along with coaching services. Sixth, the app solely provides virtual fitness game services. Seventh, the app exclusively offers sports event booking, live streaming, and participation services. Eighth, the app solely provides massage services. Ninth, the app either lacks a privacy policy or its privacy policy web page fails to display effectively. By searching, analyzing, and filtering sports and health apps, we obtained the final number of sample apps used for privacy policy compliance analysis.

### Development of the Privacy Policy Compliance Indicator Scale

We constructed a privacy policy compliance indicator scale from the perspective of the scenario-based requirement [78] and the information life cycle [79] to assess the compliance between the privacy policies of sports and health apps and the PIPL and the PI Guidelines. The process of developing the privacy policy compliance indicator scale is as follows.

We first comprehensively reviewed the provisions of the PIPL and PI Guidelines and then extracted an appropriate privacy compliance evaluation legal framework applicable to sports and health apps, from which we produce level 1 evaluation indicators. According to the information life cycle, sports and health apps operate through the processes of collection, storage, usage, processing, sharing, transfer, disclosure, information consultation, suggestion, and feedback of PI [22,27]. In light of this, we argue that the legal framework is comprised of five stages: (1) the collection of PI (Articles 6, 13, 14, 17, 28, 29, and 30 of the PIPL; and Articles 2, 4, and 5 of the PI Guidelines); (2) the storage of PI (Articles 17, 19, 39, and 40 of the PIPL; and Article 6 of the PI Guidelines); (3) the use of PI (Articles 6, 14, 15, 16, 24, 45, 46, 47, 48, 49, and 50 of the PIPL; and Articles 7 and 8 of the PI Guidelines); (4) the entrusted processing, the sharing, the transfer, and the disclosure of PI (Articles 21, 22, 23, 26, 27, 38, and 39 of the PIPL; and Article 9 of the PI Guidelines); (5) the consultation and feedback on PI (Articles 50, 57, and 65 of the PIPL; and Article 10 of the PI Guidelines). In accordance with this legal framework, we set 5 level 1 evaluation indicators: the collection of PI, the storage of PI, the use of PI, the entrusted processing, the sharing, the transfer, and the disclosure of PI, and the consultation and feedback on PI.

To conduct a more precise evaluation of the privacy policy, we further divide the 5 level 1 evaluation indicators into 37 level 2 evaluation indicators, thus establishing our privacy policy compliance indicator scale (Table 1). The level 1 evaluation indicators include PI collection, PI storage, PI usage, PI entrusted processing, sharing, transferring, and disclosing, and PI security and feedback. Specifically, the level 1 evaluation indicator of PI collection comprises 9 level 2 evaluation indicators. They are the collection subject, policy update, app scope, collection type, collection purpose, processing rule, authorization for general information, authorization for sensitive information, and exceptions for explicit authorization. Another level 1 evaluation indicator of PI storage consists of 4 level 2 evaluation indicators: storage time, storage place, PI deidentification, and sensitive information storage. We further break down the level 1 evaluation indicator of PI usage into 14 level 2 evaluation indicators, including PI access control, deidentified display and use, purpose restrictions for PI usage, authorization for the usage purpose change, constraints of automatic decision-making, query right, correction right, deletion right, copy right, account cancelation right, withdraw or change authorization, asking response right, deceased user rule, and complaint mechanism. Concerning the level 1 evaluation indicator of PI entrusted processing, sharing, transferring, and disclosing, we further categorize it into 5 level 2 evaluation indicators: requirements for entrusted controller processing PI, reasons for sharing, transferring, and disclosing PI, security measures of sharing, transferring, and disclosing PI, special circumstances without consent, and cross-border transmission requirements. The last level 1 evaluation indicator is the PI security and feedback, which encompasses 5 level 2 evaluation indicators of security incident response mechanism, security event notification mechanism, feedback channel, feedback period, and external dispute resolution mechanisms.

**Table 1.** Level 1 and level 2 evaluation indicators of privacy policy compliance of sports and health apps.

Level 1 evaluation indicator	Level 2 evaluation indicator
PI^a^ collection	Collection subject Policy update Application scope Collection type Collection purpose Processing rule Authorization for general information Authorization for sensitive information Exceptions for explicit authorization
PI storage	Storage time Storage place PI deidentification Sensitive information storage
PI usage	PI access control Deidentified display and use Purpose restrictions for PI usage Authorization for the usage purpose change Constraints of automatic decision-making Query rightCorrection right Deletion rightCopy right Account cancelation right Withdraw or change authorization Asking response right Deceased user rule Complaint mechanism
PI entrusted processing, sharing, transferring, and disclosing	Requirements for entrusted controller processing PI Reasons for sharing, transferring, and disclosing PI Security measures of sharing, transferring, and disclosing PI Special circumstances without consent Cross-border transmission requirements
PI security and feedback	Security incident response mechanism Security event notification mechanism Feedback channel Feedback period External dispute resolution mechanisms

a PI: personal information.

The PIPL and PI Guidelines are of significant importance for the protection of personal data and privacy. These provisions are legally mandatory rules that must be strictly adhered to. Therefore, we treat all 5 level 1 evaluation indicators equally and assign them a value of 1. It means that if these 37 level 2 evaluation indicators are accurately and effectively stated in the privacy policy of the sports and health app, they are assigned a value of 1, and if not, they are assigned a value of 0.

After classifying the level 1 evaluation indicators and applying the scoring rule, we got a total score (37 points) for each app’s privacy policies. Then, the final score is calculated on a 100-point scale. This means each app’s privacy policy score (on a 37-point scale) is converted to a 100-point scale to determine the final score. The evaluation results were further divided into 4 levels: excellent for a compliance score above 90 points, good for a compliance score in the range of 80‐90 points, qualified for a compliance score in the range of 60‐79 points, and unqualified for a compliance score below 60 points. The privacy policy compliance tiering of sports and health apps is illustrated in Table 2.

Finally, we calculated and summarized the score of each level 2 evaluation indicator for all sample apps. From August 21 to December 25, 2025, 2 independent raters (RG and FC) participated in the scoring process. This process was divided into 2 stages. To ensure reliability and consistency of the scoring results, in the initial scoring stage, all raters independently evaluated 30 randomly selected apps (10.5% of the total), achieving a correlation coefficient of 0.983 (*P*<.001) among different raters within the same group, indicating that the evaluations were closest to consistency. After evaluation, the raters gathered together to discuss the differences and reasons for the initial scoring and ultimately formed an almost consistent scoring standard. In this way, subjectivity in scoring is limited, although not eliminated. In the second scoring stage, we divided the sample apps into 2 groups of 143 each. Each rater randomly selects a group of apps to score independently.

**Table 2.** The privacy policy compliance tiers of sports and health apps.

Final score for apps	Privacy policy compliance tiers
≥90	Excellent
80-90	Good
60-80	Qualified
<60	Unqualified

## Results

### Sample Collection

From August 2, 2025, we conducted the collection, selection, evaluation, and analysis of the privacy policies of the sports and health apps. We gathered an initial set of 714 sports and health apps from the Android App Store and Apple App Store.

We collected 328 sports and health apps from the Android App Store. After excluding 118 apps due to reasons such as irrelevance to sports and health management services, the final valid count was 210 apps. The reasons and numbers for the 118 excluded apps are as follows: (1) 11 apps were excluded for providing only diet control and fat loss services, (2) 5 apps were excluded for providing only body data collection and testing services, (3) 27 apps were excluded for offering solely exercise instruction and learning services, (4) 17 apps were excluded for providing only virtual gaming services, (5) 35 apps were excluded for offering only sports event booking and live streaming services, (6) 5 apps were excluded for providing only sports venue booking services, (7) 16 apps were excluded for other reasons, and (8) 1 app was excluded because its privacy policy web page could not be accessed.

We collected 386 sports and health apps from the Apple App Store. After excluding 273 apps unrelated to sports and health management services, 113 valid apps remained. The reasons and quantities for excluding the 113 apps are as follows: (1) 28 apps were excluded for providing only dietary control and fat loss services; (2) 45 apps were excluded for offering solely psychological counseling and mental health guidance; (3) 36 apps were excluded for providing only sleep issue monitoring and sleep aid services; (4) 44 apps were excluded for offering exclusively physical data collection and health monitoring services; (5) 3 apps were excluded for solely providing exercise instruction and learning services; (6) 1 app was excluded for solely providing virtual gaming services; (7) 11 apps were excluded for solely providing massage and relaxation services; (8) 10 apps were excluded for solely providing traditional Chinese medicine consultation and treatment services; (9) 59 apps were excluded for other reasons including menstrual cycle prediction and care, vaccination, parenting, etc; (10) 5 apps were excluded for failing to open their privacy policy webpage; and (11) 31 apps were excluded for lacking a privacy policy. Therefore, we excluded 391 apps whose functions were not centered around sports. The reasons and number of apps excluded are listed in Table 3; the names, reasons, and number of apps excluded are presented in Multimedia Appendix 1. Ultimately, we collected 323 valid sports and health apps (the characteristics of the 323 valid sports and health apps are illustrated in Multimedia Appendix 2). The characteristics of the 323 valid sports and health apps are presented in Table 4.

**Table 3.** The reasons and number of apps excluded.

Reasons for apps excluded	Apps excluded (n=391), n
Dietary control	40
Mental health	45
Sleep monitoring	36
Body monitoring	49
Physical education	30
Video games	18
Massage services	11
Traditional Chinese medicine diagnosis and treatment	10
Sports events	35
Venue booking	5
Other reasons (menstrual care, childcare, sports knowledge dissemination, etc)	75
Unable to open the webpage	6
No privacy policy	31

**Table 4.** The characteristics of the 323 valid sports and health apps.

Characteristics	Apps (n=323), n (%)
Apps source	
Android App Store	210 (65)
Apple App Store	113 (35)
Number of Android apps downloads	
≤10,000	46 (21.9)
10,000-100,000	42 (20)
100,000-10,00,000	57 (27.1)
1,000,000-1,00,00,000	43 (20.5)
10,000,000-1,00,000,000	18 (8.6)
>10,00,00,000	4 (1.9)
Age-based app classification in Android apps	
≥3	90 (42.8)
≥8	18 (8.6)
≥12	30 (14.3)
≤16	25 (11.9)
≥18	47 (22.4)
Age-based app classification of Apple apps	
≥4	76 (67.3)
≥9	3 (2.6)
≥12	13 (11.5)
≤17	21 (18.6)
App rating of Android apps	
<1	60 (28.6)
1-2	38 (18.1)
2-3	32 (15.2)
3≤n-4	37 (17.6)
4-5	25 (11.9)
5	18 (8.6)
App rating of Android apps	
<1	2 (1.8)
12	2 (1.8)
23	10 (8.8)
34	14 (12.4)
45	81 (71.7)
5	4 (3.5)
App rating of total apps	
1	62 (19.2)
1-2	40 (12.4)
2-3	42 (13)
3-4	51 (15.8)
4-5	106 (32.8)
5	22 (6.8)

**Figure 1.**
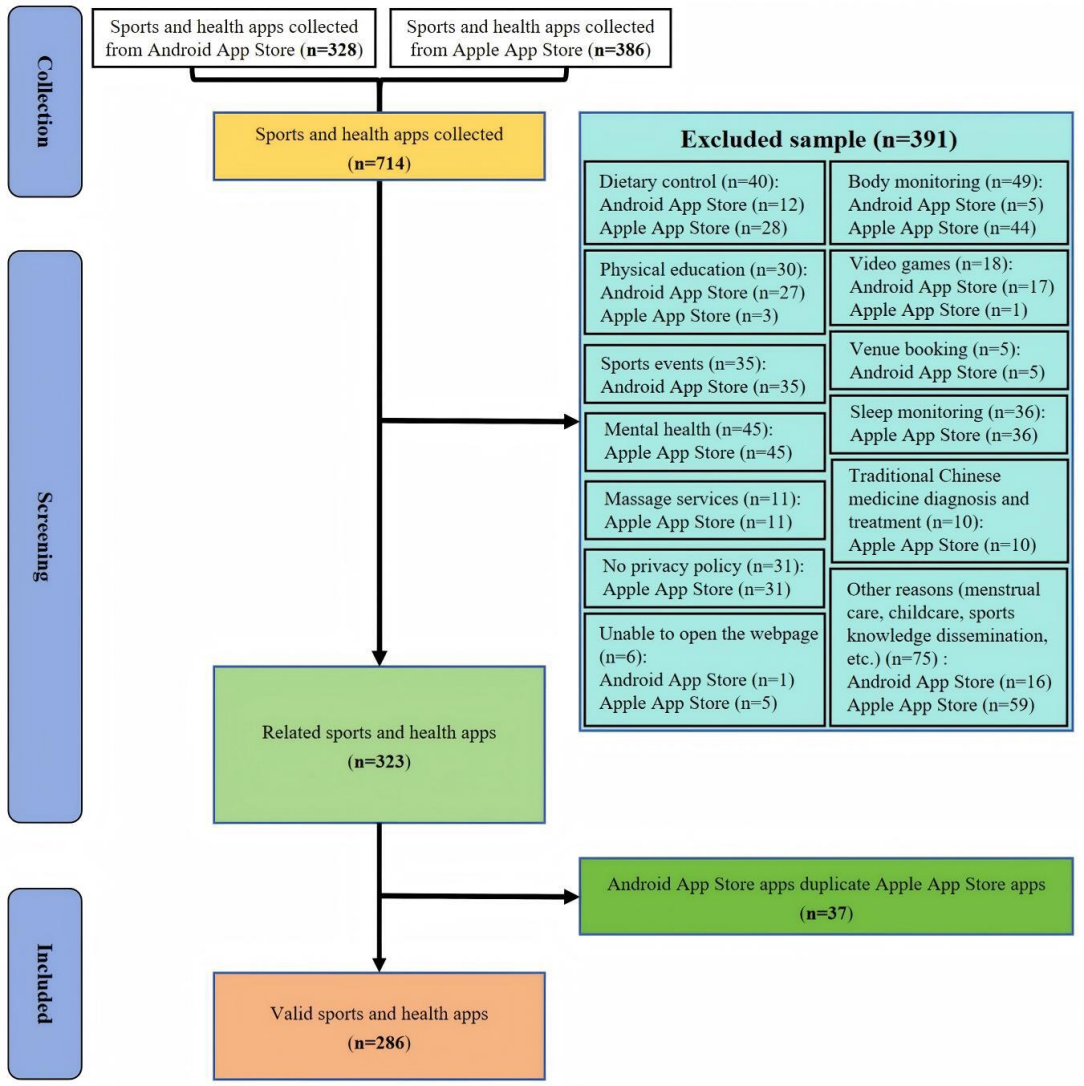
Flow diagram of the privacy policy collection and identification process.

After carefully verifying the app names and company names of 323 valid sports and health apps, we found that among the 210 valid Android apps and 113 valid Apple iOS apps, 37 apps belonged to the same company and shared identical privacy policies. Therefore, we only need to count the privacy policies of duplicate apps from 1 operating system’s app store. Since accessing privacy policies on Apple devices is more user-friendly than on Android devices, the privacy policies for these 37 duplicate apps were collected from the Apple App Store and included in the total count of apps collected from the Apple App Store. We subsequently removed these duplicate apps from the Android App Store, resulting in a final count of 286 apps. The methods and procedures for collecting, selecting, and excluding sample apps are presented in Figure 1. By entering the introduction pages of 286 sports and health apps one by one, we downloaded the privacy policy texts. After that, we thoroughly and carefully read the privacy policy documents of these apps from August 10 to August 20, 2025.

This study examined the privacy policies of 286 sports and health apps and assessed whether these privacy policies comply with the PIPL and the PI Guidelines in the Chinese Mainland. The names, compliance indicators, and scores of sports and health apps are presented in Multimedia Appendix 3. Our findings reveal a complex landscape of privacy policy compliance among sports and health apps. Among 286 sports and health apps examined, we observed a spectrum ranging from commendably high privacy compliance to moderate compliance and to a surprisingly poor compliance rate.

### Compliance Evaluation

**Figure 2.**
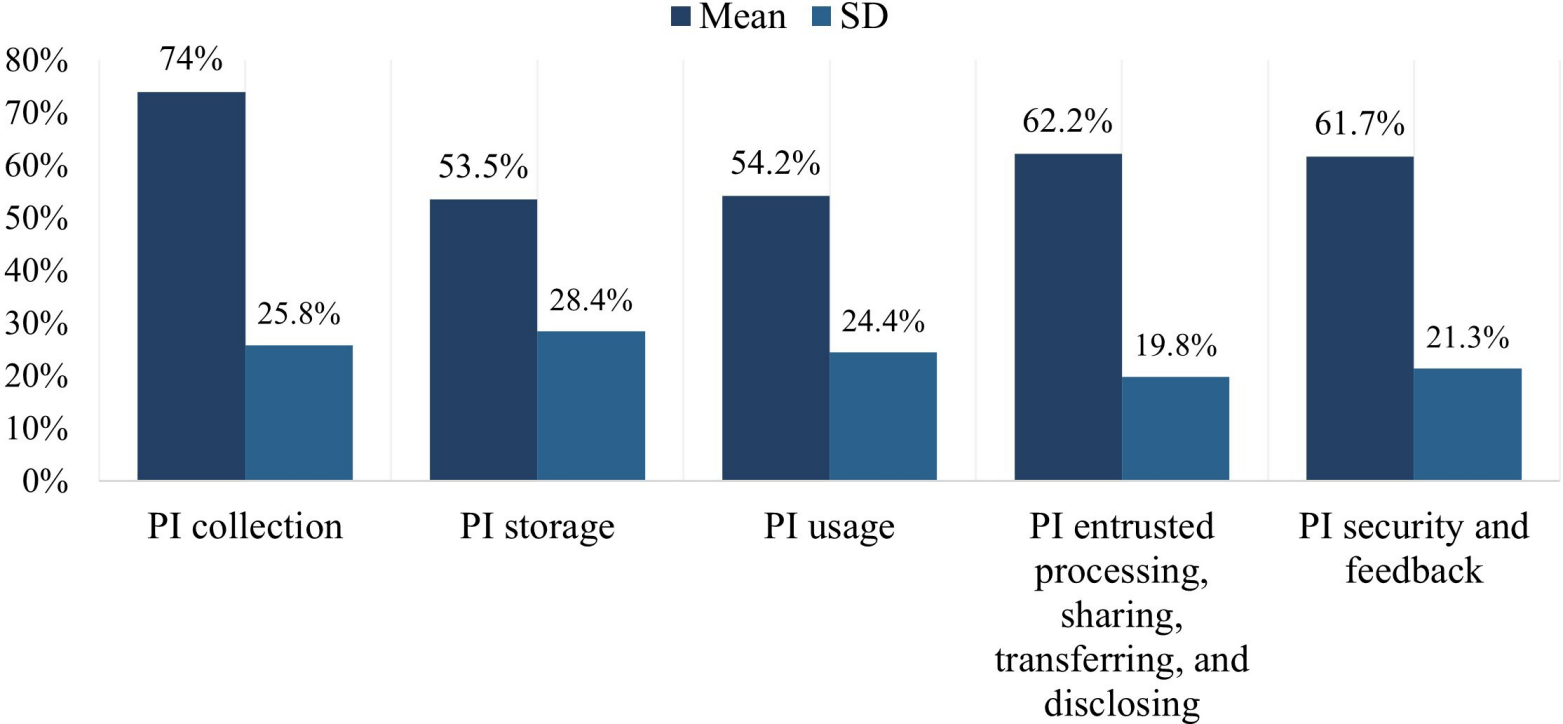
Compliance evaluation results of level 1 evaluation indicators of the apps’ privacy policies. PI: personal information.

The privacy policy compliance of 286 sports and health apps exhibits significant variation overall (Multimedia Appendix 3). The privacy policy compliance rate for level 1 evaluation indicators across these apps exhibits a tiered pattern. Among level 1 evaluation indicators, PI collection had the highest privacy policy compliance rate (mean 74%, SD 25.8%), while PI storage had the lowest compliance rate (mean 53.5%, SD 28.4%). PI usage also scored low in compliance (mean 54.2%, SD 24.4%). PI security and feedback (mean 61.7%, SD 21.3%) and PI entrusted processing, sharing, transferring, and disclosing (mean 62.2%, SD 19.8%) scored relatively higher. The overall privacy policy compliance status of these level 1 evaluation indicators is illustrated in Figure 2.

**Figure 3.**
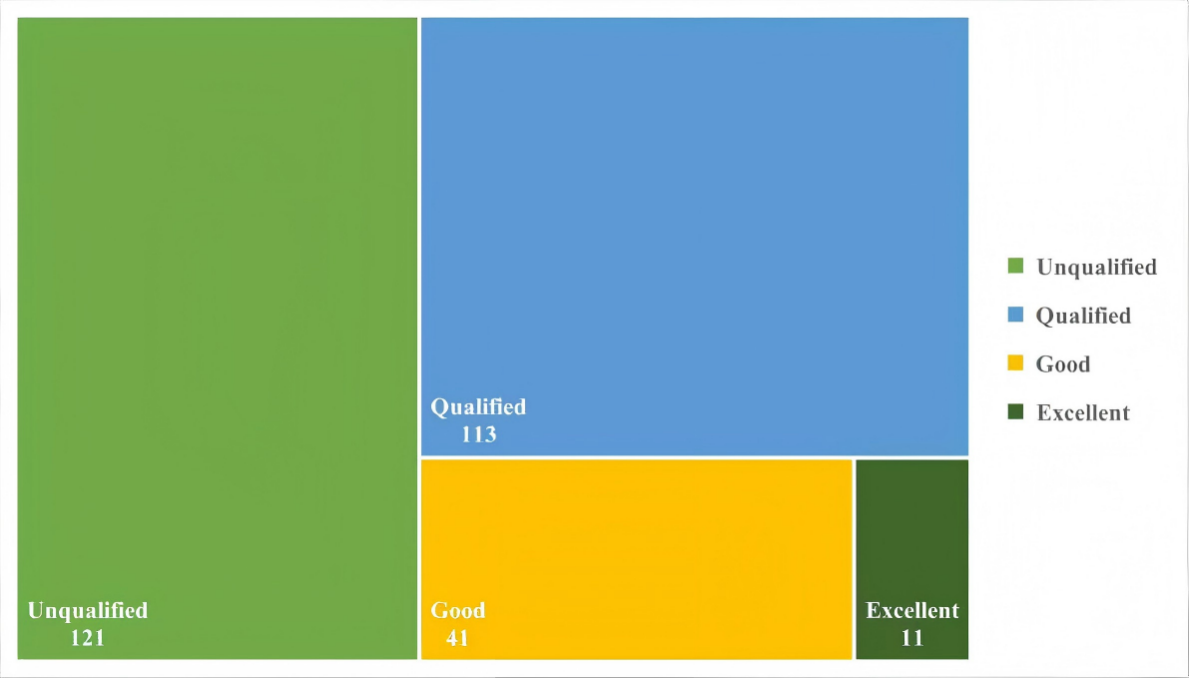
Compliance evaluation results on the overall compliance level of the apps’ privacy policies.

In general, after categorizing the privacy policy compliance scores of 286 sports and health apps into 4 levels (excellent, good, qualified, and unqualified), it was found that 3.8% (n=11) of apps achieved excellent compliance, 41 apps scored good, 113 apps scored qualified, while nearly half (n=121, 42.3%) scored unqualified. Compliance evaluation results on the overall compliance level of the apps’ privacy policies are illustrated in Figure 3.

From the perspective of the information life cycle [58], the privacy policy compliance of sports and health apps exhibits significant diversity in level 2 evaluation indicators for the information life cycle. This reflects significant variations in the implementation of privacy policy compliance among sports and health apps, with some rules being poorly enforced. The level 2 evaluation indicators’ scores of sports and health apps’ privacy policy compliance are reflected in Table 5.

**Table 5.** Level 2 evaluation indicators of privacy policy compliance score.

Level 1 and level 2 indicator	Mean (SD)
PI^a^ collection	74% (25.8%)
Collection subject	97.2% (16.5%)
Policy update	42.7% (49.5%)
Application scope	82.9% (37.7%)
Collection type	99% (10.2%)
Collection purpose	96.2% (19.3%)
Processing rule	83.2% (37.4%)
Authorization for general information	83.6% (37.1%)
Authorization for sensitive information	29.4% (45.6%)
Exceptions for explicit authorization	52.4% (50%)
PI storage	53.5% (28.4%)
Storage time	69.6% (46.1%)
Storage place	78% (41.5%)
PI deidentification	52.4% (50%)
Sensitive information storage	14% (34.7%)
PI usage	54.2% (24.4%)
PI access control	67.5% (46.9%)
De-identified display and use	34.6% (47.7%)
Purpose restrictions for PI usage	77.6% (41.8%)
Authorization for the usage purpose change	53.5% (50%)
constraints of automatic decision-making	9.4% (29.3%)
Query right	80.1% (40%)
Correction right	70.6% (45.6%)
Deletion right	79.7% (40.3%)
Copy right	46.5% (50%)
Account cancelation right	61.4% (48.8%)
Withdraw or change authorization	75.2% (43.3%)
Asking response right	45.1% (49.8%)
Deceased user rule	5.2% (22.3%)
Complaint mechanism	51.7% (50.1%)
PI entrusted processing, sharing, transferring, and disclosing	62.2% (19.8%)
Requirements for entrusted controller processing PI	52.4% (50%)
Reasons for sharing, transferring, and disclosing PI	91.6% (27.8%)
Security measures of sharing, transferring, and disclosing PI	63.6% (48.2%)
Special circumstances without consent	65.4% (47.7%)
Cross-border transmission requirements	37.8% (48.6%)
PI security and feedback	61.7% (21.3%)
Security incident response mechanism	68.5% (46.5%)
Security event notification mechanism	55.6% (49.8%)
Feedback channel	93.4% (24.9%)
Feedback period	55.6% (49.8%)
External dispute resolution mechanisms	35.3% (47.9%)

a PI: personal information.

**Figure 4.**
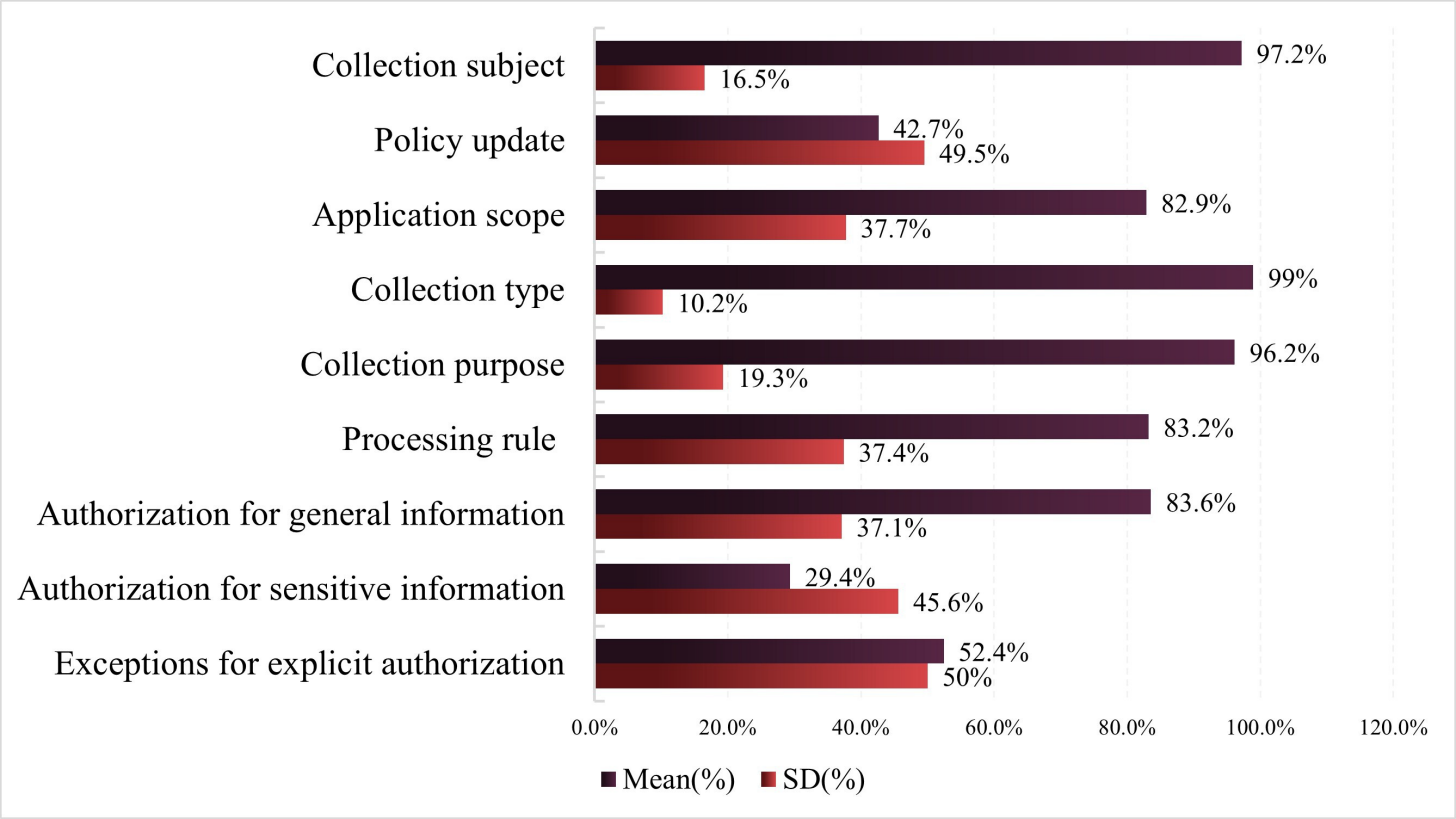
Privacy policy compliance evaluation results of personal information collection.

For level 1 evaluation metric PI collection, PI collection demonstrated the highest level of privacy policy compliance (mean 74%, SD 25.8%) (Figure 4). This indicates that sports and health apps generally prioritize compliance in PI collection, informing users about the methods and purposes of personal information gathering, thereby fundamentally ensuring users’ right to know regarding the collection of their PI. This promotes users’ understanding of how their PI is used and enhances the transparency, integrity, and security of personal data usage within sports and health apps. The level 1 evaluation indicators PI collection showed significant differences in privacy policy compliance scores across its 9 level 2 evaluation indicators. Collection type demonstrated the highest privacy policy compliance (mean 99%, SD 10.2%), indicating that sports and health apps place strong emphasis on disclosing the types of PI collected to users, ensuring users are aware that their data, particularly sensitive data, have been gathered. Sports and health apps also exhibit high privacy policy compliance rates for collection subject (mean 97.2%, SD 16.5%) and collection purpose (mean 96.2%, SD 19.3%). This indicates that the vast majority of such apps clearly state the name of the data controller and the purpose of data collection within their privacy policies. Informing users about the collection subject and collection purpose enables them to understand who collects their PI and for what processing activities it is used, thereby assisting users in future legal actions regarding PI breaches. The compliance rate for application scope (mean 82.9%, SD 37.7%), processing rule (mean 83.2%, SD 37.4%), and authorization for general information (mean 83.6%, SD 37.1%) all exceeded 80%. Policy update (mean 42.7%, SD 49.5%) and exceptions for explicit authorization (mean 52.4%, SD 50%) showed lower compliance rates, approaching half. The privacy policy compliance rate for authorization for sensitive information was the lowest (mean 29.4%, SD 45.6%), indicating that the vast majority of sports and health apps lack effective provisions in their privacy policies regarding consent mechanisms for collecting sensitive PI. Since sports and health apps inevitably collect users’ sensitive PI, the absence of consent mechanisms for such data collection leaves personal sensitive information highly vulnerable and susceptible to leakage.

**Figure 5.**
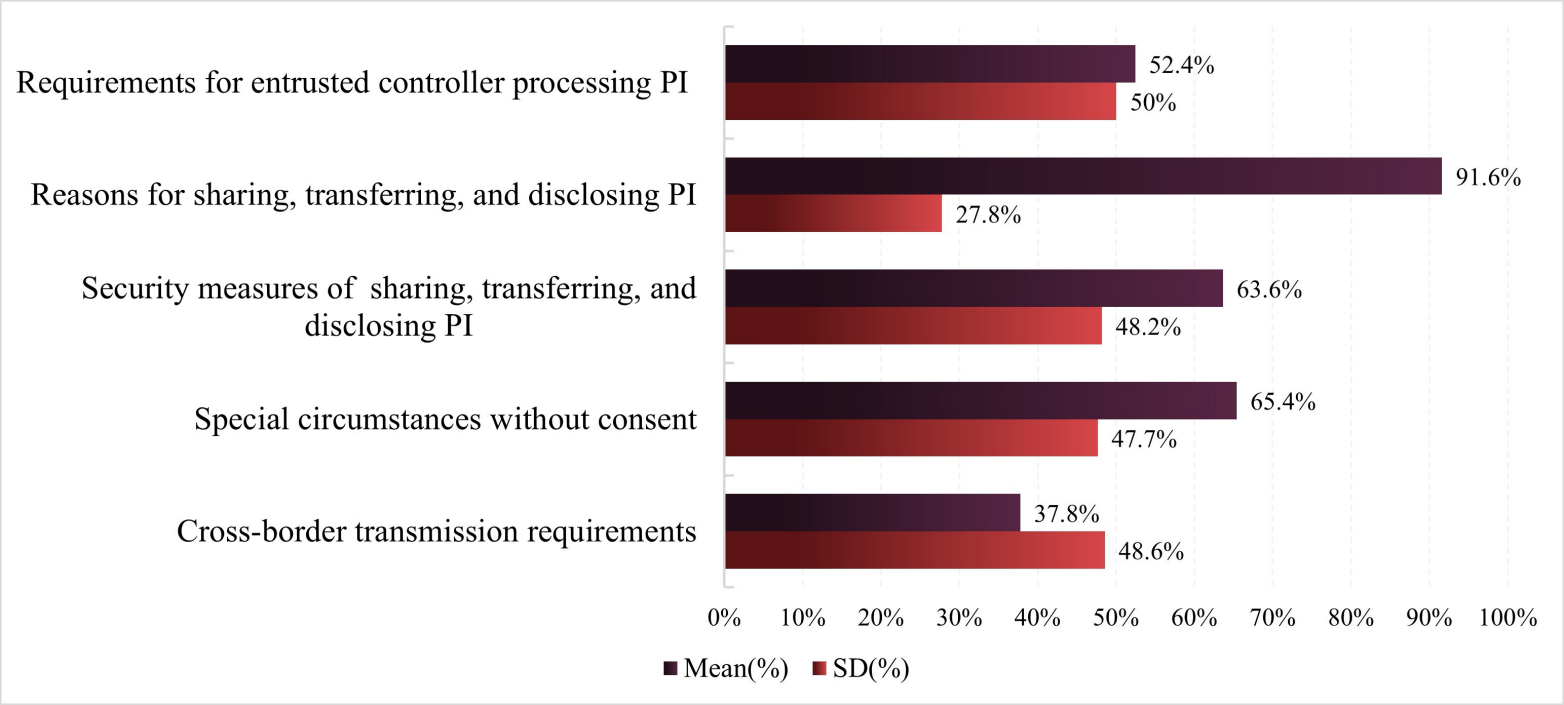
Privacy policy compliance evaluation results of personal information entrusted processing, sharing, transferring, and disclosing. PI: personal information.

The level 1 evaluation indicator PI entrusted processing, sharing, transferring, and disclosing also demonstrated high privacy policy compliance (mean 62.2%, SD 19.8%) (Figure 5). Except for cross-border transmission requirements (mean 37.8%, SD 48.6%), the privacy policy compliance rate for level 2 evaluation indicators under level 1 PI entrusted processing, sharing, transferring, and disclosing exceeded 50%. The privacy policy compliance rate for reasons for sharing, transferring, and disclosing PI was the highest (mean 91.6%, SD 27.8%), indicating that sports and health apps highly prioritize explaining these reasons to users. This assists in safeguarding users’ right to informed consent during the sharing, transferring, and disclosing of PI. The compliance rates of privacy policies for sports and health apps in the requirements for entrusted controller processing PI (mean 52.4%, SD 50%), security measures for sharing, transferring, and disclosing PI (mean 63.6%, SD 48.2%), and special circumstances without consent (mean 65.4%, SD 47.7%) all exceed one-half.

**Figure 6.**
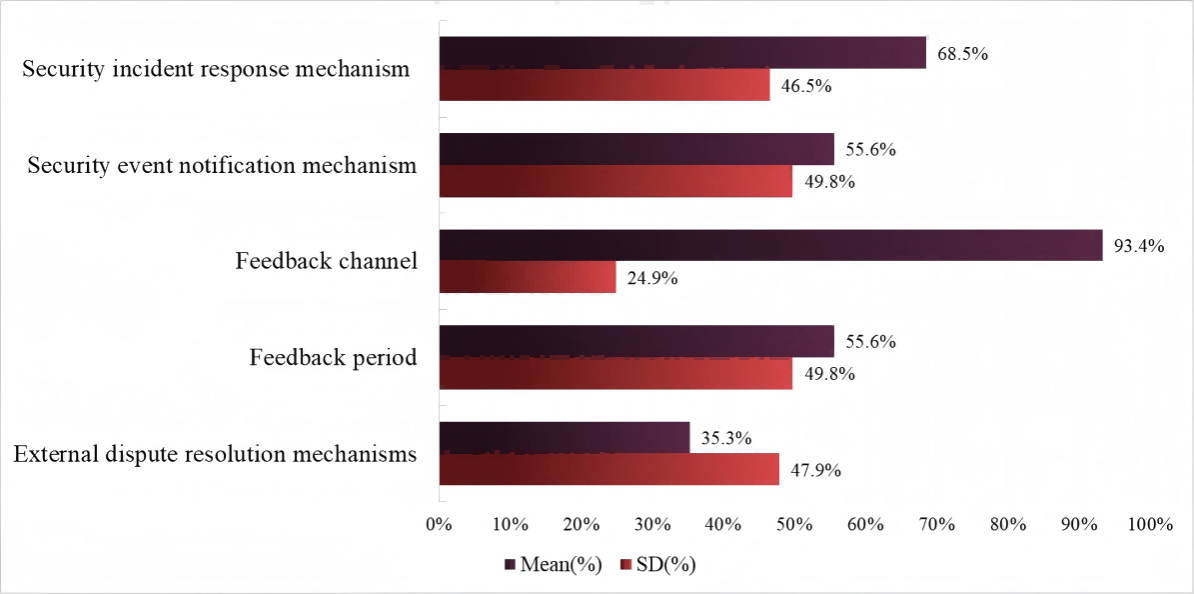
Privacy policy compliance evaluation results of personal information security and feedback.

Regarding level 1 evaluation indicators, PI security and feedback, the privacy policy compliance rate of sports and health apps in PI security and feedback exceeds half (mean 61.7%, SD 21.3%) (Figure 6). The privacy policy compliance score for the level 2 evaluation indicator feedback channel is the highest (mean 93.4%, SD 24.9%), reflecting that sports and health apps pay great attention to providing users with effective feedback channels in case of disputes regarding the collection, use, and processing of PI. In terms of privacy policy compliance, more than half of the sports and health apps meet the level 2 evaluation indicators: security incident response mechanism (mean 68.5%, SD 46.5%), security event notification mechanism (mean 55.6%, SD 49.8%), and feedback period (mean 55.6%, SD 49.85%). This indicates that over 50% of sports and health apps have established PI security protection mechanisms and security incident reporting procedures, ensuring the safety of users’ PI and their right to be informed in the event of information leakage. The compliance of external dispute resolution mechanisms in privacy policies is very poor, with one-third of sports and health apps not having established external resolution mechanisms for PI disputes, which means they do not clearly inform users that they can sue in court if a dispute occurs.

**Figure 7.**
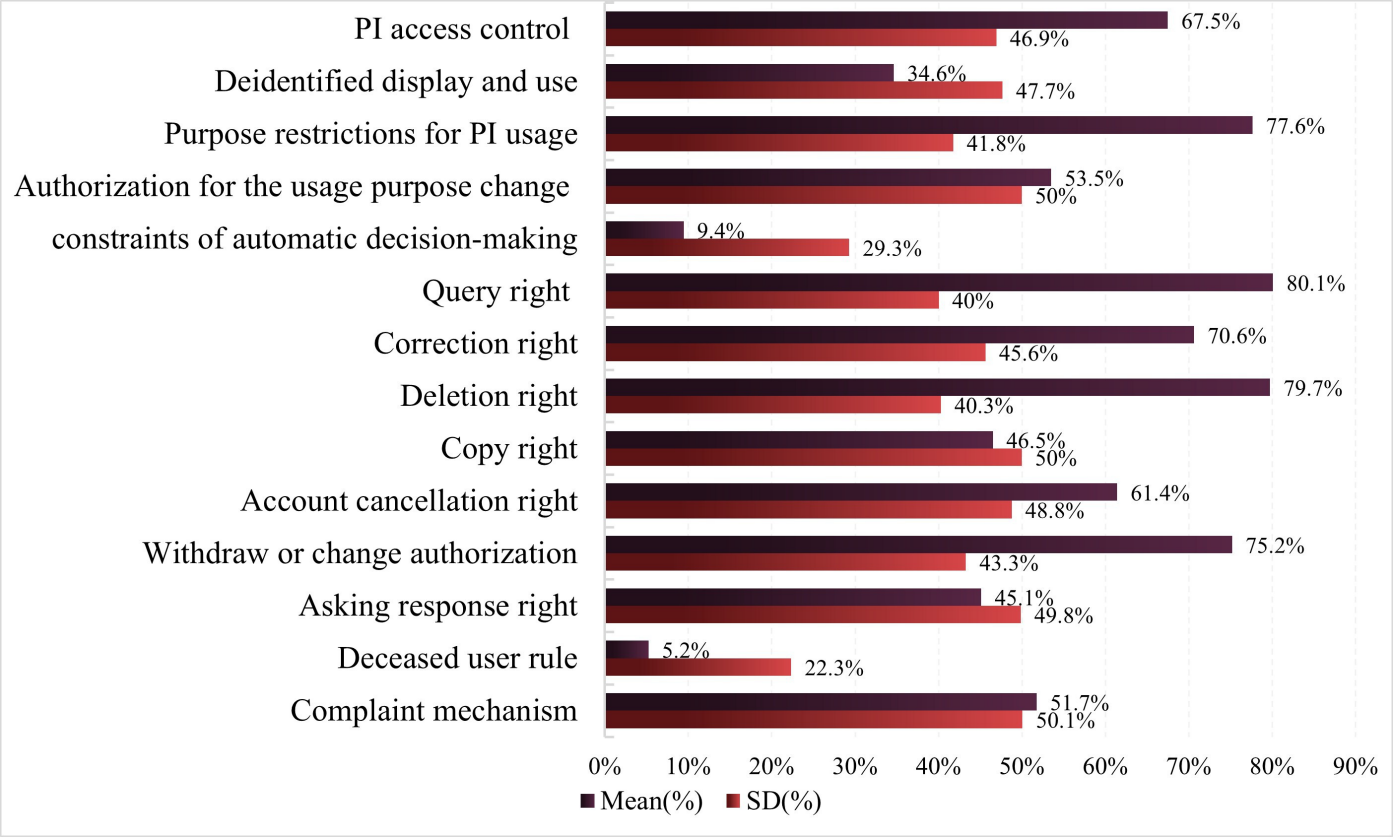
Privacy policy compliance evaluation results of personal information usage. PI: personal information.

The level 1 evaluation indicator PI usage is relatively low (mean 54.2%, SD 24.4%) for privacy policy compliance. This indicates that nearly half of the sports health apps do not strictly enforce the protective regulations for PI usage (Figure 7). Sports and health apps generally provide good protection for users’ PI rights, with the compliance rate for the query right being the highest (mean 80.1%, SD 40.0%). Compliance rates for the correction right (mean 70.6%, SD 45.6%), deletion right (mean 79.7%, SD 40.3%), account cancelation right (mean 61.4%, SD 48.8%), and withdraw or change authorization (mean 75.2%, SD 43.3%) also exceed half, indicating that the vast majority of sports and health apps can effectively protect users’ basic PI rights. However, in terms of user personal information rights, the privacy compliance rates for copyright (mean 46.5%, SD 50%) and asking response right (mean 45.1%, SD 49.8%) are relatively poor, especially for the deceased user rule, which has the worst privacy compliance (mean 5.2%, SD 22.3%). On the other hand, the privacy compliance rates for level 2 evaluation indicators such as PI access control (mean 67.5%, SD 46.9%) and purpose restrictions for PI usage (mean 77.6%, SD 41.8%) are relatively good. The privacy policy compliance rate for authorization for the usage purpose change (mean 53.5%, SD 50%) and the complaint mechanism (mean 51.7%, SD 50.1%) have both exceeded half. Only one-third of sports and health apps meet privacy policy compliance requirements in deidentified display and use (mean 34.6%, SD 47.7%). It is particularly noteworthy that the compliance rate of privacy policies regarding constraints of automatic decision-making is very low (mean 9.4%, SD 29.3%), indicating that the vast majority of sports and health apps do not have restrictions on the automatic decision-making mechanism. This is detrimental to users’ ability to decide how their PI is used and processed, leading to negative impacts on the security of PI and users’ peace of mind [80].

**Figure 8.**
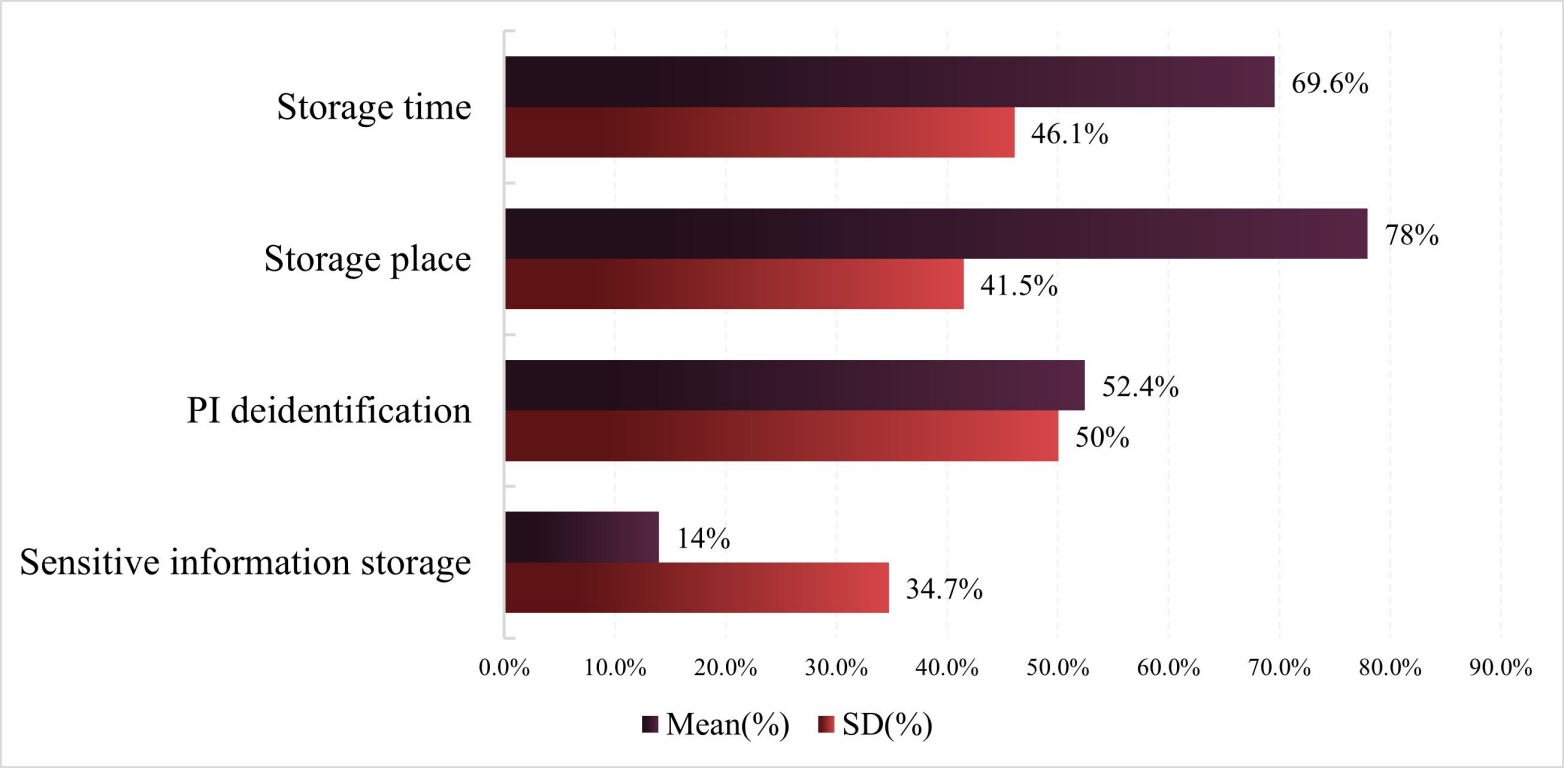
Privacy policy compliance evaluation results of personal information storage. PI: personal information.

The compliance rate of privacy policies for sports and health apps regarding the level 1 evaluation indicator PI storage is the lowest (mean 53.5%, SD 28.4%), with about half of the apps failing to meet the compliance requirements for PI storage (Figure 8). The compliance rate of privacy policies for sports and health apps regarding the level 2 evaluation indicator storage place is the highest (mean 78%, SD 41.5%), while the compliance extent for storage time (mean 69.6%, SD 46.1%) and PI deidentification (mean 52.4%, SD 50%) also performs relatively well. However, the compliance rate of privacy policies for sensitive information storage is the worst among sports and health apps, indicating that the vast majority of apps do not have specific provisions and settings for the storage of users’ sensitive PI, which is detrimental to the protection of users’ sensitive PI and data security.

## Discussion

### Principal Findings

We developed a privacy policy evaluation scale by integrating scenario-based contextual analysis theory and information life cycle theory, grounded in the provisions of the PIPL and PI Guidelines, to assess privacy policies of mobile sports and health apps. Our defined context encompasses scenarios where users use these apps to record physical activity data, exercise routes, and fitness instructions, all directly related to physical exercise.

This study’s findings demonstrate that 286 evaluated apps in the Chinese Mainland are failing to meet core PI protection requirements, with critical gaps in the overall compliance of the 286 apps, policy update, sensitive PI protection, key user rights, PI transferring, and PI security management.

First, our analysis indicates that the overall compliance level of privacy policies among 286 sports and health apps is not high. The compliance levels varied (mean 61%, SD 24.3%), with a minority of apps demonstrating strong compliance while others showed lower compliance. Sports and health apps with low compliance primarily violate the PIPL and PI Guidelines, which share common principles with many other jurisdictions. Furthermore, low privacy policy compliance of apps can have several direct and indirect negative consequences for users, including security risks, loss of control, and intrusive marketing. This indicates the need to strengthen PI regulatory oversight for sports and health apps and establish standardized, actionable practices.

Second, data analysis suggests that PI collection has the highest level of compliance. According to Article 14 of the PIPL, if the collection purpose, method, or types are changed, a separate consent shall be obtained from users. However, data analysis indicates that compliance rates regarding privacy policy updates are unsatisfactory, with only 122 out of 286 apps adhering to this requirement. Indeed, many app developers operate under the assumption that continued use equates to user consent, rather than proactively seeking explicit approval. This lack of compliance undermines both user dignity and security, increasing the risk of data misuse and breaches. To address this, we argue that apps should instead secure explicit consent through clear pop-up notifications, much like during the initial sign-up process. Users should be required to actively confirm their agreement; otherwise, access to the app’s services should be limited or denied.

Third, we found that the privacy policies of sports and health apps exhibit significant shortcomings in protecting sensitive PI. It should be noted that the PIPL (especially Chapter II, Section 2) and the Guidelines (especially Article 5.4) provide special protection for the collection of sensitive PI. Substantively, Article 28 of the PIPL specifies the substantive conditions for processing sensitive PI, namely, PI processors can process sensitive PI only when there is a specific purpose and when it is of necessity, under the circumstances where strict protective measures are taken. Procedurally, Articles 29 and 30 of the PIPL and Article 5.4b of the PI Guidelines require that a processor processing sensitive PI shall notify the individual of the necessity of processing their sensitive PI and the impact it has on their rights and interests,” and that sensitive PI processors must obtain informed, voluntary, specific, clear, unequivocal, and separate consent from PI subjects. The substantive and procedural requirements have also been highlighted by the Notice of the Ministry of Industry and Information Technology on Further Enhancing the Service Capacity of Mobile Internet Applications [81]. However, our data analysis shows that the compliance rate of sensitive PI protection (mean 29.4%, SD 45.6%) is significantly lower than that of general PI protection (mean 83.6%, SD 37.1%). This disparity not only indicates how inadequate the present security measures are for protecting sensitive PI, but it also continuously lowers users’ awareness of security when providing sensitive PI. Additionally, it is also important to note that although a total of 40 apps referenced sensitive PI storage, none comply with the storage requirements of the PI Guidelines, which mandate encryption (Article 6.3.a) and, in principle, the elimination of original personal biometric data (Article 6.3.c), including samples and photographs. In the 40 apps, one just lists the types of sensitive PI, such as personal identity information and biometric information. The other apps fail to explicitly state the protection measures for sensitive PI; instead, their statements imply that adequate protection measures have been taken and that they would do their best to protect sensitive PI. This reveals a serious security concern, because if sensitive PI is leaked, tampered with, or used illegally, it may endanger the user’s personal or property safety.

Fourth, our analysis indicates that comprehensive compliance with the protection of users’ PI rights remains weak. In terms of user rights, although most apps effectively list a series of user rights, including query rights, correction rights, deletion rights, withdraw or change authorization rights, and account cancellation rights, less than half of the apps give users the right to obtain a copy of PI and ask for a response. Notably, only 27 of the 286 apps provide users with the right to refuse using automated decision-making mechanisms. Currently, personalized recommendation technologies and artificial intelligence technologies are advancing rapidly. Automated decision-making mechanisms based on algorithms have been widely adopted in the operation of sports and health apps. The vast majority of these apps do not grant users the right to opt out of automated decision-making processes. This results in the infringement of users’ autonomy over the collection, use, and processing of their personal information, violating the principles of legitimacy and transparency enshrined in PIPL. Consequently, users may be misled in their app usage, experience improper collection of their information, and face frequent disruption from app notifications. Fewer apps have provided deceased user rules, that is, unless otherwise arranged by the deceased before their death, their close relatives have the rights to access, copy, correct, delete, and other rights related to the deceased’s relevant PI. This will trigger disputes over the usage and inheritance of deceased individuals’ digital assets, hindering the normal operation and development of sports and health apps.

Fifth, our findings reveal that privacy policies for sports and health apps require further refinement regarding regulations on sharing, transferring, and disclosing PI. While most apps state the reasons for sharing, transferring, and disclosing PI, significantly fewer of them will take technical measures to ensure information security and obtain the user’s separate consent. Notably, even fewer apps’ privacy policies specify that when PI is shared or transferred to overseas processors, the user should be separately informed of the purpose of data export and the receiver, and the user’s authorization should be separately obtained. This is obviously lower than the standards stipulated in Article 23 of the PIPL and Article 9 of the PI Guidelines, which may damage the users’ ability to monitor the whole PI processing process, their security awareness of transferring PI, and their trust in apps, and the principle of informed consent.

Finally, our analysis reveals that sports and health apps do not provide users with clear response times for feedback. User data collection occurs in real time, and this frequent gathering inevitably leads to issues or disputes during usage. At such times, a timely and effective feedback and dispute resolution mechanism becomes crucial for resolving problems, maintaining user retention, and helping apps build a positive reputation. However, many sports and health apps merely provide feedback channels without establishing timelines for addressing concerns. This lack of accountability discourages apps from proactively and effectively resolving feedback issues and disputes. Additionally, many sports and health apps lack robust PI security safeguards and reporting mechanisms, making personal data vulnerable to infringement and posing serious risks of data breaches. This will lead to a decline in users’ trust in the app’s privacy protection, making users more cautious about providing personal information, especially sensitive personal information, and may lead to users uninstalling the app.

### Recommendations

The study’s findings collectively demonstrate that 286 evaluated apps are failing to meet core PI protection requirements. Critical gaps in PI protection are primarily attributed to 3 factors. First, there is a deficiency of awareness among users regarding PI protection, reducing incentives for app developers to prioritize privacy. Second, existing PI guidelines lack legally binding force, resulting in inconsistent adherence and voluntary compliance. Third, insufficient regulatory oversight and weak enforcement mechanisms fail to deter noncompliant practices. These factors collectively undermine the effectiveness of current PI protection frameworks, highlighting the need for targeted action from 3 stakeholders: users, regulators, and legislators. We argue that only through coordinated action can the app ecosystem close the compliance gaps identified in this study, reduce PI protection risks, and restore user trust in digital services.

First, to strengthen app privacy compliance, it is essential to enhance users’ PI rights awareness [82]. We argue that interventions can be scenario-embedded and accessible. For example, app developers are encouraged to integrate snapshots of PI rights into app onboarding when users consent to privacy policies, paired with visual resources that translate legal jargon into simple guidance [83]. This ensures users learn rights when they interact with PI-related features, not just through disconnected policy texts. Additionally, official complaint channels should be easily accessible to hold apps accountable for noncompliance, which can drive industry-wide improvement [84].

Second, the regulatory mechanism for sports and health apps should be standardized. Under the current legal framework, the regulatory mechanism for apps can be characterized as self-regulation by app operators, with government oversight as a supplementary measure. Moreover, in terms of the institutional framework in the Chinese Mainland, regulators are dispersed across many different government departments, resulting in a lack of enthusiasm for law enforcement and of technical capacity for supervision. As a result, the regulation of apps in practice is inadequate. In this context, we recommend creating a unique data regulator to assess and oversee the privacy protection of apps and raising the industry’s entry barrier through the issuance of compliance identification or certifications [64]. This regulator can strengthen enforcement of low-compliance areas, such as sensitive PI storage and policy updates, through regular audits and penalties for noncompliance [63].

Finally, laws and regulations for the protection of personal health information should be improved. The Civil Code [85], the Cybersecurity Law of China [86], the Law of the People’s Republic of China on Basic Medical and Health Care and the Promotion of Health [87], and the PIPL formulate the legal framework for protecting personal health information. While these laws are comprehensive, the majority of them contain oath and principle clauses that are not applicable in specific cases. Although many of these principles and oath clauses are further crafted in the PI Guidelines, these guidelines do not have the force of law; instead, they are merely recommended standards and are not mandatory by nature. In this regard, we suggest that the legal effect of adopting the PI Guidelines should be clarified in laws and regulations. In this way, the app operator should specify in the first part of the privacy policy whether the PI Guidelines are adopted, and once adopted, they will be legally binding [66].

### Contributions

This study holds significant implications for both policymakers and scholars. First, based on the principal findings—particularly that the privacy protection requirements established by the PIPL demonstrate a higher level of compliance compared with the PI Guidelines—we propose that legislation should strengthen the binding force of the PI Guidelines, thereby providing guidance for legislative reform. Second, the current low compliance with privacy policies may be linked to inadequate regulatory enforcement. In response to the fragmented regulatory oversight in practice, we recommend the establishment of an independent regulator to enhance the effectiveness of privacy protection enforcement. Finally, this study addresses a gap in the existing literature. While existing studies have explored the relationship between health apps and privacy protection in Australia [4,88], between mHealth apps and privacy protection [89-93], and between health code apps and privacy protection in China [94,95], the legal compliance of sports and health apps with the PIPL and PI Guidelines, as well as China’s legislative framework for PI protection, was not thoroughly examined in these studies. This study fills this gap by examining the privacy policies of 286 sports and health apps across 37 evaluation dimensions and assessing whether these privacy policies comply with the PIPL and PI Guidelines.

### Limitations

However, this study also has limitations. Although we developed an indicator scale to assess the compliance level of privacy policies of the 286 sports and health apps, this evaluation process does not concern the actual implementation of different technical measures, resulting in the omission of evaluation criteria and the lack of targeted recommendations for improvement from a technical point of view [96]. For instance, an app may explicitly state in its policy that it encrypts sensitive PI during storage but may not actually implement consistent encryption protocols in its backend systems. We hope that future research can treat this study as a starting point to further analyze the processing of PI and the design and implementation of PI protection measures in the information system, by using technical methods, such as network traffic analysis or third-party data flow auditing, and adopting the research method of interviewing relevant personnel including product managers, research and development engineers, PI protection officers, legal personnel, system architects, security administrators, operation and maintenance personnel, human resources personnel, system users, and so on.

### Conclusions

The extensive usage of sports and health apps is significantly enhancing the social and public health landscape in the Chinese Mainland. Nonetheless, the security threats to PI protection arising from privacy policy compliance have not received adequate attention. This paper’s assessment of the overall compliance level of 286 sports and health apps and the individual evaluation of 37 privacy policies reveals a complex picture of PI protection. Although some apps establish commendable compliance policies, there are some shortcomings. These shortcomings not only pose security threats to app users but may also hinder operators in optimizing and developing app functionalities. In light of this, this paper puts forward suggestions for improvement from the perspectives of user awareness, regulatory enforcement, and legislative reforms.

## Supplementary material

10.2196/73651 Multimedia Appendix 1 Excluded samples.

10.2196/73651 Multimedia Appendix 2 The characteristics of the valid sports and health apps.

10.2196/73651 Multimedia Appendix 3 Names, compliance indicators, and scores of sports and health apps.

10.2196/73651 Checklist 1 PRISMA-LSR checklist.

## Abbreviations

mHealth: mobile health
PI: personal information
PIPL: Personal Information Protection Law
