# Supplementary material for: Privacy Policy Compliance of Mobile Sports and Health Apps in China: Scale Development, Data Analysis, and Prospects for Regulatory Reform
Source: JMIR Mhealth Uhealth. 2026 Feb 11;14:e73651. doi: 10.2196/73651 (PMC12893645; doi:10.2196/73651)
Supplement: Checklist 1 [file mhealth-v14-e73651-s004.pdf]

Preferred Reporting Items for Systematic reviews and Meta-Analyses extension for Living Systematic Reviews (PRISMA- LSR) Checklist

Section and topic	PRISMA 2020 checklist items*	PRISMA-LSR checklist		Reported on page #
		Items	Elements	
**Title**				
Title	1. Identify the report as a systematic review	—	• Identify the report as “living” in the title†<br>• Provide the version number	N/A<br>N/A
**Abstract**				
Abstract	2. See PRISMA 2020 for abstracts checklist	—	• Indicate whether the LSR is being retired from the living mode after the publication of the current version, if applicable	N/A
**Introduction**				
Rationale	3. Describe the rationale for the review in the context of existing knowledge	—<br>—	• Justify the use of the living mode<br>• Cite the preceding version of the LSR, if applicable	3-7<br>N/A
Objectives	4. Provide an explicit statement of the objective(s) or question(s) the review addresses	—	• Describe and justify any changes since the preceding version to the review's objective(s) or question(s)	7-8
**Methods**				
Living mode parameters	—	L1. Specify the living mode parameters	• Specify the planned schedule of the search for each source (eg, at a prespecified interval, following predefined triggers)	8
			• Specify the planned schedules for the remaining steps of the systematic review (eg, at a prespecified interval, following predefined triggers), if applicable. The remaining steps include screening, data collection, risk of bias assessment, analysis, certainty of evidence assessment, publication	8
			• Specify the plan for retirement from the living mode (eg, based on a prespecified timeline, following predefined triggers), if there is one. If there is no such plan, indicate so	N/A

Section and topic	PRISMA 2020 checklist items*	PRISMA-LSR checklist		Reported on page #
		Items	Elements	
Eligibility criteria	5. Specify the inclusion and exclusion criteria for the review and how studies were grouped for the syntheses	—	—	8-9
Information sources	6. Specify all databases, registers, websites, organisations, reference lists, and other sources searched or consulted to identify studies. Specify the date when each source was last searched or consulted	—	—	13-17
Search strategy	7. Present the full search strategies for all databases, registers and websites, including any filters and limits used	—	—	8
Selection process	8. Specify the methods used to decide whether a study met the inclusion criteria of the review, including how many reviewers screened each record and each report retrieved, whether they worked independently, and if applicable, details of automation tools used in the process	—	—	13
Data collection process	9. Specify the methods used to collect data from reports, including how many reviewers collected data from each report, whether they worked independently, any processes for obtaining or confirming data from study investigators, and if applicable, details of automation tools used in the process	—	• Describe whether the review team updated or planned to update collected data for a previously included study when relevant	13
Data items	10a. List and define all outcomes for which data were sought. Specify whether all results that were compatible with each outcome domain in each study were sought (eg, for all measures, time points, analyses), and if not, the methods used to decide which results to collect	—	—	9-12
	10b. List and define all other variables for	—	—	9-12; 33-

Section and topic	PRISMA 2020 checklist items*	PRISMA-LSR checklist		Reported on page #
		Items	Elements	
	which data were sought (eg, participant and intervention characteristics, funding sources). Describe any assumptions made about any missing or unclear information			34
Study risk of bias assessment	11. Specify the methods used to assess risk of bias in the included studies, including details of the tool(s) used, how many reviewers assessed each study and whether they worked independently, and if applicable, details of automation tools used in the process	—	• Describe whether the review team updated or planned to update risk of bias information for a previously included study when relevant	13
Effect measures	12. Specify for each outcome the effect measure(s) (eg, risk ratio, mean difference) used in the synthesis or presentation of results	—	—	12-13
Synthesis methods	13a. Describe the processes used to decide which studies were eligible for each synthesis (eg, tabulating the study intervention characteristics and comparing against the planned groups for each synthesis—item 5)	—	—	N/A
	13b. Describe any methods required to prepare the data for presentation or synthesis, such as handling of missing summary statistics, or data conversions	—	—	9
	13c. Describe any methods used to tabulate or visually display results of individual studies and syntheses	—	—	12-13
	13d. Describe any methods used to synthesise results and provide a rationale for the choice(s). If meta-analysis was performed, describe the model(s), method(s) to identify the presence and extent of statistical heterogeneity, and software package(s) used	—	• Report any analytical methods applied specifically because of the living mode	12-13

Section and topic	PRISMA 2020 checklist items*	PRISMA-LSR checklist		Reported on page #
		Items	Elements	
	13e. Describe any methods used to explore possible causes of heterogeneity among study results (eg, subgroup analysis, meta-regression)	—	—	N/A
	13f. Describe any sensitivity analyses conducted to assess robustness of the synthesised results	—	—	N/A
Reporting bias assessment	14. Describe any methods used to assess risk of bias due to missing results in a synthesis (arising from reporting biases)	—	—	N/A
Certainty assessment	15. Describe any methods used to assess certainty (or confidence) in the body of evidence for an outcome	—	—	N/A
Changes to the methods	—	L2. Describe changes to the methods	• Describe and justify any changes since the preceding version to the methods (items L1, 5-15)	N/A
			• If there are no changes to the methods, indicate so	N/A
			• Indicate whether the changes to the methods were applied to previously included studies	N/A
**Results**				
Study selection	16a. Describe the results of the search and selection process, from the number of records identified in the search to the number of studies included in the review, ideally using a flow diagram	—	• Describe what triggered the current version, if applicable	13-17
			• Ideally, use a flow diagram to illustrate the results of the search and selection processes in the different versions of the review using one of the LSR tailored flow diagrams	17
	16b. Cite studies that might appear to meet the inclusion criteria, but which were excluded, and explain why they were excluded	—	—	N/A
Study characteristics	17. Cite each included study and present its characteristics	—	—	N/A

Section and topic	PRISMA 2020 checklist items*	PRISMA-LSR checklist		Reported on page #
		Items	Elements	
Risk of bias in studies	18. Present assessments of risk of bias for each included study	—	—	N/A
Results of individual studies	19. For all outcomes, present, for each study: (a) summary statistics for each group (if appropriate) and (b) an effect estimate and its precision (eg, confidence or credible interval), ideally using structured tables or plots	—	—	N/A
Results of syntheses	20a. For each synthesis, briefly summarise the characteristics and risk of bias among contributing studies	—	—	N/A
	20b. Present results of all statistical syntheses conducted. If meta-analysis was done, present for each the summary estimate and its precision (eg, confidence or credible interval) and measures of statistical heterogeneity. If comparing groups, describe the direction of the effect	—	• Report results of any analytical methods applied specifically because of the living mode	18-27
	20c. Present results of all investigations of possible causes of heterogeneity among study results	—	—	N/A
	20d. Present results of all sensitivity analyses conducted to assess the robustness of the synthesised results	—	—	N/A
Reporting biases	21. Present assessments of risk of bias due to missing results (arising from reporting biases) for each synthesis assessed	—	—	N/A
Certainty of evidence	22. Present assessments of certainty (or confidence) in the body of evidence for each outcome assessed	—	—	N/A
Changes to the results	—	L3. Describe changes to the	• Indicate the studies that were included since the preceding version (related to PRISMA 2020	N/A

Section and topic	PRISMA 2020 checklist items*	PRISMA-LSR checklist		Reported on page #
		Items	Elements	
		results	item 17)	
			•Describe and justify the changes since the preceding version in the eligibility status of any study (ie, excluding a previously included study, including a previously excluded study; related to PRISMA 2020 item 16)	N/A
			• Describe any other consequential changes since the preceding version to the results	N/A
			• If there are no changes to the results, indicate so	N/A
**Discussion**				
Discussion	23a. Provide a general interpretation of the results in the context of other evidence	—	—	27-31
	23b. Discuss any limitations of the evidence included in the review	—	—	33-34
	23c. Discuss any limitations of the review processes used	—	• Discuss any limitations related to the living mode	33-34
	23d. Discuss implications of the results for practice, policy, and future research	—	• Describe any changes since the preceding version to the implications of the results for practice, policy, and future research	31-33
			• Describe and justify any planned changes to review methods in upcoming review versions	N/A
			• Indicate and justify whether the LSR is being retired from the living mode following the publication of the current version, if applicable	N/A
**Other information**				
Registration and protocol	24a. Provide registration information for the review, including register name and registration number, or state that the review was not registered	—	—	N/A

Section and topic	PRISMA 2020 checklist items*	PRISMA-LSR checklist		Reported on page #
		Items	Elements	
	24b. Indicate where the review protocol can be accessed, or state that a protocol was not prepared	—	—	N/A
	24c. Describe and explain any amendments to information provided at registration or in the protocol‡	—	—	N/A
Support	25. Describe sources of financial or non-financial support for the review, and the role of the funders or sponsors in the review	—	• Describe the sources of financial or non-financial support and the roles of funders or sponsors in each of the versions of the LSR	35
Competing interests	26. Declare any competing interests of review authors	—	• Describe the competing interests of review authors and how they were managed for each of the versions of the LSR	35
Availability of data, code and other materials	27. Report which of the following are publicly available and where they can be found: template data collection forms; data extracted from included studies; data used for all analyses; analytic code; any other materials used in the review		• Describe any changes since the preceding version to the accessibility of data, code and materials	35
Authors and their roles for each version of the LSR	—	L4. Provide the list of authors and their roles for each version of the LSR	—	35

*Elements of the PRISMA 2020 checklist are not listed here. However, they should also be reported if applicable.

†While PRISMA 2020 item 1 included an additional (ie, non-essential) element to report whether "the review is a continually updated ('living') systematic review," in PRISMA-LSR, this is an essential element.

‡Item 24c of the PRISMA 2020 checklist is replaced by item L2 of the PRISMA-LSR checklist.
